# Clinical and computed tomography features of extended-spectrum β-lactamase-producing *Klebsiella pneumoniae* liver abscess

**DOI:** 10.1186/s12879-020-05142-z

**Published:** 2020-06-15

**Authors:** Yue Ren, Hairui Wang, Zhihui Chang, Zhaoyu Liu

**Affiliations:** Department of Radiology, Shengjing Hospital of China Medical University, NO. 36, Sanhao Street, Heping District, Shenyang, 110004 China

**Keywords:** *Klebsiella pneumoniae*, Liver abscess, Extended-spectrum β-lactamase, CT

## Abstract

**Background:**

*Klebsiella pneumoniae* (KP) is the primary pathogen associated with pyogenic liver abscesses (PLAs). Moreover, there has been an increase in the proportion of extended-spectrum beta-lactamase (ESBL)-producing KP. However, the clinical and computed tomography (CT) features of liver abscesses caused by ESBL-producing KP have not been separately described. We aimed to compare the clinical and CT features present in patients with ESBL-producing and non-ESBL-producing KP as well as to determine the risk factors for ESBL-producing KP liver abscesses (KPLAs).

**Methods:**

We performed a retrospective analysis of data obtained from the medical records of patients with a first episode of KPLA admitted to Shengjing Hospital of China Medical University between May 2015 and May 2019. We compared the clinical and CT features between patients with ESBL-producing and non-ESBL-producing KPLA.

**Results:**

We enrolled 100 patients with KPLA (14 and 86 in the ESBL-producing and non-ESBL-producing groups, respectively). There was no significant between-group difference in the proportion of patients with comorbid diabetes (71.43% vs. 66.2%, *p* = 0.086). The ESBL-producing KPLA group had a greater proportion of patients with a history of biliary disease (78.57% vs. 26.74%, *p* < 0.001) and gastrointestinal malignancy (50% vs. 6.98%, *p* < 0.001). Multivariate regression analysis showed that a history of biliary disease was an independent risk factor for ESBL-producing KPLA. Compared with the non-ESBL-producing KPLA group, the ESBL-producing KPLA group had a significantly higher intensive care unit (ICU) admission rate (28.57% vs. 2.33%, *p* < 0.001). All ESBL-producing KP isolates were susceptible to carbapenems and amikacin. Only the presence of multiloculation on CT was found to be significantly different between the groups (50% vs. 82.56%, *p* = 0.012).

**Conclusions:**

The presence of biliary disease was an independent risk factor for ESBL-producing KPLA. Patients with ESBL-producing KPLA had a higher ICU admission rate, with only half of patients having evidence of multiloculation on CT.

## Background

*Klebsiella pneumoniae* (KP) is a key pathogen in nosocomial and community-acquired infections [1–3]. Moreover, it is the main pathogen associated with pyogenic liver abscesses (PLAs) [4–7]. Several studies have shown that the occurrence of PLA, caused by extended-spectrum β-lactamase–producing (ESBL-producing) KP, has been increased worldwide [8–11]. One report showed that the prevalence of ESBL-producing KP liver abscesses (KPLAs) has increased from 1.64% in 2001 to 14.29% in 2011 in Singapore [10]. The main treatment methods for PLA include antibiotics, percutaneous drainage or aspiration, and surgery, as appropriate [12–15]. Bacterial culture and drug sensitivity analysis allow for the determination of the involved pathogenic bacteria and effective antibiotics. However, a proportion of patients presents with negative bacterial cultures. Moreover, preparing bacterial cultures are time-consuming [13, 16]. In the clinical treatment of PLAs, antibiotics are only administered empirically before the drug sensitivity results are available. ESBL production by pathogens often causes poor efficacy of empirically selected antibiotics [17–19]. On the other hand, early and effective antibiotic therapy decreases the mortality rate of KPLA [13]. Therefore, analysing the clinical and computed tomography (CT) features of PLAs caused by ESBL-producing KP may assist in determining the typical characteristics of ESBL-producing KPLA and enable a faster identification of these ESBL-producers.

In this study, we performed a retrospective analysis of data obtained from the medical records of patients with a first episode of KPLA. We aimed to compare the clinical and CT features of patients with ESBL-producing and non-ESBL-producing KPLA, and to determine the risk factors for ESBL-producing KPLA.

## Methods

### Study design

**Fig. 1:**
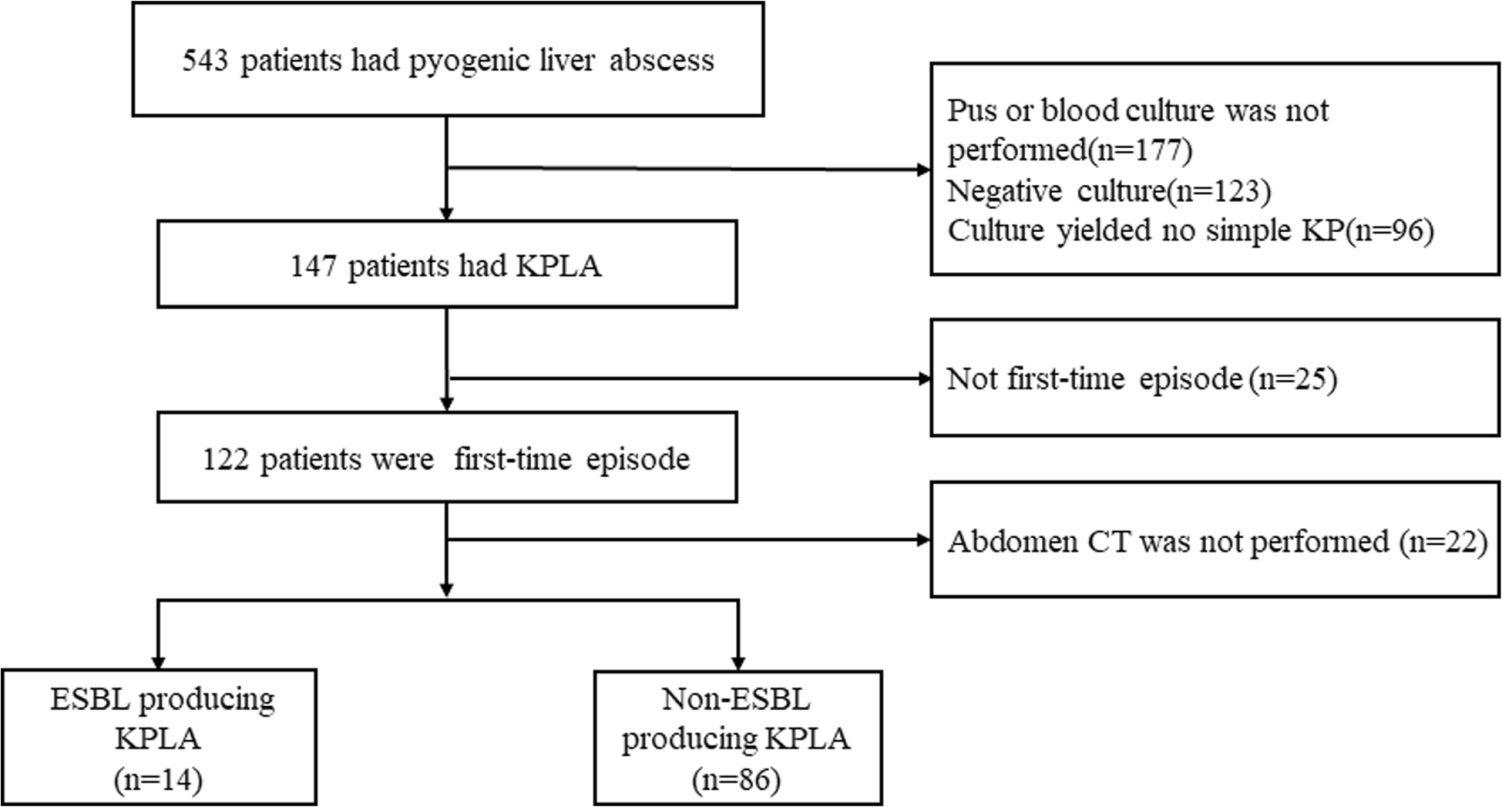
Flowchart of patients enrolment

We obtained the medical records of patients with a first episode of KPLA admitted to Shengjing Hospital of China Medical University between May 2015 and May 2019. This is a 6750-bed university hospital with 4.46 million outpatient and emergency visits annually. The Ethics Committee at Shengjing Hospital of China Medical University approved this retrospective study. The enrolment process of patients is shown in Fig. 1.

### Patients and definitions

The inclusion criteria were as follows: (1) liver abscess symptoms, including fever, chills, and pain in the region of the liver; (2) abdominal CT examination showing liver abscess lesions; and (3) pus or blood bacterial culture indicating KP. The exclusion criteria were as follows: (1) recurrent liver abscess, defined as the presentation of recurrent typical clinical and imaging findings after complete treatment of the first PLA episode; (2) other concomitant bacterial infections, such as the simultaneous isolation of KP and other bacteria on blood or pus cultures; and (3) patients with incomplete or insufficient data.

### Data collection

We collected the following patient information: age, sex, the underlying cause (history of biliary disease, history of gastrointestinal malignancy, and diabetes), clinical symptoms and signs (fever, chills, nausea, vomiting, etc.), concurrent endophthalmitis, treatment methods, antibiotic resistance status, and whether the patient was transferred to the intensive care unit (ICU). A history of biliary disease included that of hepatolithiasis, choledocholithiasis, cholelithiasis, and other benign biliary diseases but not cholangiocarcinoma. A history of gastrointestinal malignancy included that of primary and recurrent gastrointestinal malignancies (including cholangiocarcinoma), regardless of whether the patient underwent the relevant surgery, radiotherapy, or chemotherapy.

### CT features

**Fig. 2:**
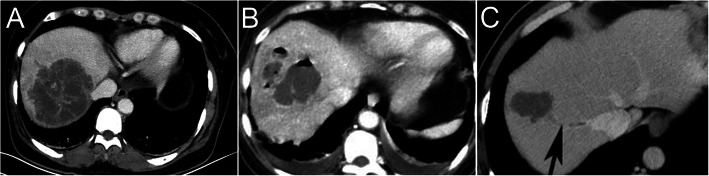
CT features of KPLA. **a**, Contrast-enhanced CT showing a multilocular liver abscess. Pus bacterial culture and drug sensitivity results are indicative of KP (non-ESBL-producing). **b**, CT showing a liver abscess with visible gas in the abscess cavity. Bacterial culture and drug sensitivity results are indicative of KP (ESBL-producing). **c**, CT showing a unilocular liver abscess, while a venous-phase contrast-enhanced scan shows a right venous filling defect. Blood bacterial culture and drug sensitivity results are indicative of KP (non-ESBL-producing). CT, computed tomography; ESBL-producing KPLA, extended-spectrum β-lactamase–producing *Klebsiella pneumoniae* liver abscess

We only reviewed the contrast-enhanced CT images obtained before drainage of the liver abscess for the purpose of this study. The CT scanning equipment used, scope, and methods are consistent with our previous studies [8, 20]. Two radiologists with more than 8 years of work experience reviewed the scans and reached a consensus. The following features were recorded: (a) number of abscesses (single or multiple); (b) liver involvement (unilobar right or left or bilobar); (c) maximal abscess diameter, with the largest abscess measured when there were multiple abscesses; (d) unilocular or multilocular (presence of ≥1-mm-thick septations, Fig. 2a); (e) gas within the abscess cavity (Fig. 2b); (f) thrombophlebitis (hypodense filling defects in the contrast-enhanced hepatic veins, their tributaries, and/or the inferior vena cava) as shown in Fig. 2c; and(g) spontaneous rupture of the abscess (based on CT and clinical symptoms).

### Microbiologic data

All strains were tested for drug sensitivity using the VITEK-compact automatic microbiological analysis system (France BioMérieux) and AST-N334 card. Minimum inhibitory concentration values of 20 representative antibiotics were detected using the micro-release method; these were amikacin, gentamicin, levofloxacin, ciprofloxacin, imipenem, meropenem, aztreonam, ampicillin, ampicillin/sulbactam, cefepime, cefuroxime, cefoxitin, cefazolin, ceftriaxone, ceftazidime, tetracycline, nitrofurantoin, chloramphenicol, cefmetazole, and trimethoprim/sulfamethoxazole. Analyses of drug sensitivity test results were performed using a drug sensitivity test interpretation system. Phenotypic confirmation of ESBL was performed using the double-disk diffusion method in our clinical microbiology laboratories, following Clinical and Laboratory Standards Institute guidelines [18].

### Statistical analysis

SPSS software (Version 22; SPSS Inc., Chicago, USA) was used for statistical data analysis. The measurement data are expressed as mean ± standard deviation (SD), and count data are expressed as the number of cases and percentages. For data with a normal distribution, the χ^2^ test or t test was used; if the data were non-normally distributed, the non-parametric test method was used. The underlying diseases related to the pathogenesis of the liver abscess that had statistical differences in the univariate analysis (*P* < 0.1) were analysed by multivariate logistic regression. *P* < 0.05 was considered statistically significant.

## Results

### Between-group comparison of clinical features

We enrolled 100 patients with KPLA (14 in the ESBL-producing group and 86 in the non-ESBL-producing group). All patients in the ESBL-producing group had a history of underlying disease and previous hospitalization, whereas 43 patients in the non-ESBL-producing group had no prior history of hospitalization, 19 of these 43 whom were previously healthy and had no underlying medical disease. As shown in Table 1, there was no significant between-group difference in the proportion of patients with concomitant diabetes. More patients in the ESBL-producing KPLA group had a history of biliary disease (78.57% vs. 26.74%, *p* < 0.001) and gastrointestinal malignancies (50% vs. 6.98%, *p* < 0.001). Moreover, the ESBL-producing group had a higher ICU admission rate than the non-ESBL-producing KPLA group. Furthermore, there were no significant between-group differences in the main clinical symptoms, treatment methods, and 30-day mortality. Multivariate analysis revealed that a history of biliary disease was an independent risk factor for ESBL-producing KPLA (Table 2). There were no significant between-group differences in the patients’ laboratory test results on admission (Table 3).

**Table 1:** Comparison of the general characteristics of patients with ESBL-positive and -negative KPLA

	ESBL (+) KPLA *n* = 14	ESBL (−) KPLA *n* = 86	P
**General clinical features**			
Age (mean age ± SD)	60.8 ± 8.5	59.3 ± 13.3	0.95
Male, n (%)	7 (50%)	50 (58.14%)	0.57
**Underlying cause, n (%)**			
Diabetes	10 (71.43%)	45 (66.2%)	0.086
Gastrointestinal malignancy	7 (50%)	6 (6.98%)	< 0.001
History of biliary disease	11 (78.57%)	23 (26.74%)	< 0.001
**Symptoms/signs, n (%)**			
Fever (> 38.5 °C)	13 (92.86%)	82 (95.34%)	0.693
Chills	8 (57.14%)	66 (77.74%)	0.123
Nausea and/or vomiting	6 (42.86%)	21 (24.42%)	0.152
Endophthalmitis	0 (0%)	2 (2.35%)	0.566
**Duration of antibiotic treatment (mean days ± SD)**	16.36 ± 13.92	9.25 ± 4.96	0.59
**30 days mortality, n (%)**	2 (14.29%)	2 (2.33%)	0.093
**ICU admission, n (%)**	4 (28.57%)	2 (2.33%)	< 0.001

**Table 2:** Multivariate analysis of risk factors for ESBL-producing KPLA

	β	OR (95%CI)	p
Diabetes	1.448	4.255 (0.861–21.012)	0.076
Gastrointestinal malignancy	1.494	4.456 (0.879–22.578)	0.071
Biliary disease	2.505	12.245 (2.333–64.271)	0.003

**Table 3:** Comparison of laboratory test results between patients with ESBL-producing and non-ESBL producing KPLA

	ESBL (+) KPLA *n* = 14	ESBL (−) KPLA *n* = 86	P
C-reactive protein	152.93 ± 109.21	178.59 ± 98.09	0.491
Alanine aminotransferase	175.64 ± 334.34	84.93 ± 79.17	0.747
Aspartate aminotransferase	169.14 ± 250.77	56.16 ± 47.31	0.566
Alkaline phosphatase	210.88 ± 172.72	181.74 ± 94.88	0.991
Total bilirubin	23.15 ± 23.92	17.26 ± 13.09	0.33
Albumin	30.86 ± 7.98	30.27 ± 5.26	0.724
D-dimer	1425.72 ± 447.51	1962.27 ± 347.40	0.353
PT	14.35 ± 2.72	15.07 ± 10.40	0.799
White blood cell	13.38 ± 9.72	12.80 ± 4.89	0.731
Percentage of neutrophils	80.12 ± 11.82	80.90 ± 12.97	0.824
Haemoglobin	106.14 ± 28.05	118.45 ± 19.21	0.135
Platelet	192.64 ± 111.98	238.10 ± 135.77	0.240

### KP resistance to antibiotics in both groups

The patients in this study were all treated with antibiotics. We treated them empirically with carbapenems and adjusted the antibiotic based on to the results of the drug sensitivity tests. The resistance rates for ampicillin were 100% for ESBL producing KP and 83.7% for non-ESBL-producing KP. No isolate of this study was resistant to carbapenems. Notably, all ESBL-producing KP and non-ESBL-producing KP were susceptible to amikacin. In general, the ESBL-producing KP showed higher resistance rates to most antibiotics than the non-ESBL-producing KP (Table 4).

**Table 4:** Drug resistance rates to antibiotics by ESBL-producing and non-ESBL producing KP strains

Antibiotics	ESBL (+) *n* = 14	ESBL (−) *n* = 86	P
Amikacin	0	0	1
Gentamicin	50%	1.16%	< 0.001
Levofloxacin	21.43%	3.49%	0.009
Ciprofloxacin	42.86%	2.33%	< 0.001
Imipenem	0	0.00%	1
Meropenem	0	0.00%	1
Aztreonam	71.43%	2.33%	< 0.001
Ampicillin	100%	83.72%	0.105
Ampicillin/Sulbactam	57.14%	1.16%	< 0.001
Cefepime	50%	3.49%	< 0.001
Cefuroxime	35.71%	2.33%	< 0.001
Cefoxitin	7.14%	2.33%	0.33
Cefazolin	92.86%	6.98%	< 0.001
Ceftriaxone	71.43%	1.16%	0.001
Ceftazidime	50%	0.00%	< 0.001
Tetracycline	21.43%	5.81%	0.047
Nitrofurantoin	21.43%	5.81%	0.047
Chloramphenicol	85.71%	8.14%	< 0.001
Cefmetazole	0	0.00%	1
Trimethoprim/sulfamethoxazole	50%	6.98%	< 0.001

### Between-group comparison of CT features of liver abscesses

We found that 50 and 82.56% of the abscesses caused by ESBL-producing and non-ESBL producing KP, respectively, were multilocular with the between-group difference being significant (*P* < 0.05). There was no between-group difference in the remaining CT features (Table 5).

**Table 5:** CT features of ESBL-producing and non-ESBL-producing KPLA

	ESBL (+) *n* = 14	ESBL (−) *n* = 86	P
Position			0.247
Left lobe	6 (42.86%)	10 (11.63%)	
Right lobe	7 (50%)	68 (79.07%)	
Bilateral	1 (7.14%)	8 (9.30%)	
Single	10 (71.43%)	61 (70.93%)	0.621
Maximum diameter, cm	8.17 ± 2.79	7.93 ± 2.32	0.426
Multilocular	7 (50%)	71 (82.56%)	0.012
Gas-containing	5 (35.71%)	15 (17.44%)	0.115
Spontaneous rupture	0	8 (9.3%)	0.268
Thrombophlebitis	3 (21.43%)	4 (4.65%)	0.229

## Discussion

This study revealed an association between a history of biliary disease and gastrointestinal malignancies and ESBL-producing KPLA. Multivariate analysis indicated that a history of biliary disease was an independent risk factor for ESBL-producing KPLA, which is consistent with the findings of Shi et al. [21]. However, their study included liver abscesses caused by various Enterobacterales species, while our study only included KP, which is the primary pathogen associated with PLAs. Bacterial invasion via the biliary tract is among the main infectious routes in liver abscess; moreover, a PLA is a common complication after cholelithiasis surgery [22–25]. Many studies indicated that acquisition of blaCTX-M genes and resulting CTX-M enzyme production is the main cause of resistance to aztreonam and third generation cephalosporins in KP and *Escherichia coli* [26, 27]. A serum epidemiological study isolated 592 strains (62.1%) of KP from stool samples from 954 healthy adults in Asian countries [28]. It is worth noting that new hypervirulent variants of *Klebsiella pneumoniae* (hvKP) are emerging globally, most of which exhibit antimicrobial susceptibility. In an analysis of KP strains in hospitalized patients, 33% were hvKP, of which about 17% expressed ESBL, and this data increased year by year [29]. Under pathological conditions, KP crosses the intestinal mucosal barrier and enters the liver via the portal system, which causes a PLA [30]. Patients with a history of biliary disease (especially biliary-enteric anastomosis) or gastrointestinal malignancies are more susceptible to liver infections by intestinal bacteria due to intestinal mucosal damage [4, 8, 31, 32]. Moreover, these patients often undergo surgical interventions, chemotherapy, radiotherapy, and antibiotic therapy; this could negatively influence the gut flora and the immune system and my enhance the risk of ESBL-producing KPLA.

Previous studies have reported that diabetes is an independent risk factor for KPLA [33–35]. However, in our study, both univariate and multivariate analyses showed that diabetes was not associated with ESBL-producing KPLA. Patients with comorbid diabetes have a weaker immune function, which makes them more susceptible to serious infections, including pneumonia, meningitis, and endophthalmitis when infected by KP [35]. Therefore, administering a higher dose of antibiotics increases the risk of being infected with ESBL-producing KP [27]. However, one study has reported that diabetes could have a protective factor against ESBL-producing KP [21]. These partly contrasting findings in the above mentioned and or study may result from different KP populations with varying degrees of virulence. Molecular analyses on genetic relationship and known virulence genes of KPLA isolates were not performed; this is a limitation of the present study.

Contrast-enhanced CT is an effective method for the diagnosis of a PLA [20, 36]. CT revealed multiloculation in only half of the patients with ESBL-producing KPLA, which was significantly lesser than those with non-ESBL-producing KPLA. KPLA has been shown to typically manifest with multiloculation and poor liquification on CT [37]. Similar characteristics were reported by Kim et al., which were referred to as the “turquoise sign” [38]. In our study, 78% of the patients had evidence of multiloculation on CT. This could be attributed to the formation of granulation or congested liver tissues that are non-necrotic. The underlying pathological mechanism could be associated with the high virulence and anti-phagocytic properties of KP [39, 40]. Further studies, especially detailed molecular analyses are needed to determine why the afore mentioned signs are less common in ESBL-producing KPLA. In addition, antibiotic use may change the appearance of the liquefaction and loculation of the abscess. However, because of the retrospective nature of this study, we were unable to obtain the timeline for antibiotic administration and imaging in both groups.

KPLA is often associated with extrahepatic metastatic infections, including endophthalmitis [41]. These multi-site infections are now referred to as invasive KPLA syndrome (IKPLAS); however, its specific pathogenesis remains unclear. We previously reported that hepatic venous thrombophlebitis is an important sign of IKPLAS [8]. However, we did not find a correlation between ESBL-producing KPLA and hepatic venous thrombophlebitis. Studies have shown that hypermucoviscous KP infections induce platelet aggregation, and platelet hyperreactivity may be associated with a higher risk of vascular complications [42, 43]. Within the inflammatory microenvironment, endothelial cell activation and endothelial barrier destruction could promote bacterial migration [44, 45]. On the other hand, platelet and coagulation factor activation promotes thrombus formation. We found no correlation between ESBL-producing KPLAs and thrombophlebitis and endophthalmitis in our study. Given the small sample size, we need to be cautious about such results.

All ESBL-producing KP in the present study were susceptible to carbapenems and amikacin; this in in contrast to other studies that enrolled more patients with abdominal infection [11]. Previous studies have shown that the empiric use of carbapenem antibiotics could reduce the mortality risk associated with KPLA [17, 19]. This is especially true in patients with IKPLAS, which involves more serious conditions. The therapeutic efficacy of carbapenem antibiotics could be significantly superior to other antibiotics [3, 22], which is consistent with our findings. The carbapenem-resistant KP, which has been reported worldwide, is under scrutiny because of the few therapeutic options available for these strains [46]. To the best of our knowledge PLA caused by carbapenem-resistant KP is still very rare [22].

## Conclusions

We found an association between ESBL-producing KPLA and a history of biliary disease and gastrointestinal malignancy. Moreover, a history of biliary disease is an independent risk factor for ESBL-producing KPLA. Patients with ESBL-producing KPLA had a higher ICU admission rate, with only half showing multiloculation on CT. Therefore, it should be noted that patients with a history of biliary disease or gastrointestinal malignancy who present with a PLA are likely to have an ESBL-producing KPLA, especially if CT findings are indicative of uniloculation. Future studies with larger sample sizes are needed to confirm our findings.

## Data Availability

The datasets used and/or analysed during the current study are available from the corresponding author on reasonable request.

## Abbreviations

KP: *Klebsiella pneumoniae*
PLA: Pyogenic liver abscess
ESBL: Extended-spectrum beta-lactamase
CT: Computed tomography
KPLA: KP liver abscess
IKPLAS: Invasive KPLA syndrome
SD: Standard deviation
